# Monocyte HLA‐DR level on admission predicting in‐hospital mortality rate in exertional heatstroke: A 12‐year retrospective study

**DOI:** 10.1002/iid3.1240

**Published:** 2024-04-17

**Authors:** Fanfan Wang, Fanghe Gong, Xuezhi Shi, Jiale Yang, Jing Qian, Lulu Wan, Huasheng Tong

**Affiliations:** ^1^ The First School of Clinical Medicine Southern Medical University Guangzhou China; ^2^ Department of Intensive Care Unit General Hospital of Southern Theatre Command of PLA Guangzhou China; ^3^ Department of Neurosurgery General Hospital of Southern Theatre Command of PLA Guangzhou China

**Keywords:** exertional heatstroke, immunity, monocyte HLA‐DR, prognosis, risk factor

## Abstract

**Background:**

Exertional heatstroke (EHS), a fatal illness, pronounces multiple organ dysfunction syndrome (MODS) and high mortality rate. Currently, no ideal factor prognoses EHS. Decreased monocyte human leukocyte‐DR antigen (mHLA‐DR) has been observed in critically ill individuals, particularly in those with sepsis. While most research focus on the pro‐inflammatory response exploration in EHS, there are few studies related to immunosuppression, and no report targeted on mHLA‐DR in EHS. The present study tried to explore the prognostic value of mHLA‐DR levels in EHS patients.

**Methods:**

This was a single‐center retrospective study. Clinical data of EHS patients admitted to the intensive care unit of the General Hospital of Southern Theatre Command between January 1, 2008, and December 31, 2020, were recorded and analyzed.

**Results:**

Seventy patients with 54 survivors and 16 nonsurvivors were ultimately enrolled. Levels of mHLA‐DR in the nonsurvivors (41.8% [38.1–68.1]%) were significantly lower than those in the survivors (83.1% [67.6–89.4]%, *p* < 0.001). Multivariate logistic regression indicated that mHLA‐DR (odds ratio [OR] = 0.939; 95% confidence interval [CI]: 0.892–0.988; *p* = 0.016) and Glasgow coma scale (GCS) scores (OR = 0.726; 95% CI: 0.591–0.892; *p* = 0.002) were independent risk factors related with in‐hospital mortality rate in EHS. A nomogram incorporated mHLA‐DR with GCS demonstrated excellent discrimination and calibration abilities. Compared to the traditional scoring systems, the prediction model incorporated mHLA‐DR with GCS had the highest area under the curve (0.947, 95% CI: [0.865–0.986]) and Youden index (0.8333), with sensitivity of 100% and specificity of 83.33%, and a greater clinical net benefit.

**Conclusion:**

Patients with EHS were at a risk of early experiencing decreased mHLA‐DR early. A nomogram based on mHLA‐DR with GCS was developed to facilitate early identification and timely treatment of individuals with potentially poor prognosis.

## Graphical abstract

Predictive model superior to score systems for evaluating prognosis in exertional heatstroke patients.

## 1 INTRODUCTION

Heatstroke (HS), classified as classic heatstroke (CHS) or Exertional heatstroke (EHS), is a fatal heat‐related illness characterized by core temperature >40°C and multiple organ dysfunction syndrome (MODS), which is dominated by central nervous system (CNS) abnormalities, during passively encountering hot and humid environments or strenuous activities.^1^ During the 2003 European heat wave, HS caused about 30,000 deaths.^2^ When hypotension develops, the mortality rate in EHS is often greater than 30%.^3^ The mortality rate of HS is predicted to increase by up to 2.5 and 5 times in 2050 and 2080 as a result of the deterioration of global warming and extreme weather.^4^, ^5^ Compared to CHS, EHS, sporadic in nature, often pictures more prompt progression and pronounces multiorgan system damages.^6^ Early detection and efficient therapy are crucial for enhancing the survival of EHS patients. Nevertheless, there is currently no ideal factor for determining the prognosis of EHS.

The human leukocyte antigen(HLA)‐DR, a trustworthy marker of monocyte activating,^7^ can assist monocytes in presenting antigen signals to T cells to initiate immune reaction, which is crucial for host defense, immunological homeostasis, and immune surveillance.^8^ Low monocyte HLA‐DR (mHLA‐DR) levels have been observed in critically ill individuals with severe burns,^9^ hepatitis,^10^ pancreatitis^11^ and trauma,^12^ as well as sepsis particularly.^13^ Current research has verified that the expression of mHLA‐DR below 30% indicates that the individual is immunologically paralyzed and vulnerable to secondary infection, MODS, and even death.^14^, ^15^

The signature of the “sepsis‐like syndrome,” such as postcardiac arrest syndrome (PCAS), is systemic hyperinflammation,^16^ which may be mediated by monocytes with several pattern recognition receptors (PRRs), such as coreceptor CD14.^17^ However, another feature is the immunosuppressive phenotype, identified by the downregulation of mHLA‐DR.^16^, ^17^ HS, a “like‐sepsis” pathophysiological process,^1^ presents a more swift and intense inflammatory response and immune disorder.^6^ Patients with EHS may suffer the coexistence of hyperinflammation and immune paralysis like PCAS, which jointly drives the occurrence and development of MODS. Currently, much research focuses mainly on exploring the pro‐inflammatory response in EHS.^18^ However, there are few studies related to immunosuppression and immune paralysis, and no report targeted mHLA‐DR in EHS. Therefore, this study attempted to explore the relationship between mHLA‐DR and outcomes in EHS.

## 2 PATIENTS AND METHODS

### 2.1 Patients

From January 1, 2008 to December 31, 2020, clinical data of EHS patients were collected and investigated retrospectively at the intensive care unit (ICU) of the General Hospital of Southern Theatre Command of People's Liberation Army (PLA) of China. The EHS diagnostic criteria were as follows: (1) a history of passively encountering hot and humid environments or strenuous activities. (2) core temperature over 40°C, concomitant disorders of the central nervous system, involving encephalopathy, seizure, or coma.^3^ The study protocol was confirmed by the Medical Ethics Review Committee of the General Hospital of Southern Theater of PLA of China (No. NZLLKZ2022047), and the demand for informed authorization was eliminated. The standards for reporting of diagnostic accuracy checklist was used.^19^

### 2.2 Inclusion and exclusion criteria

**Figure 1:**
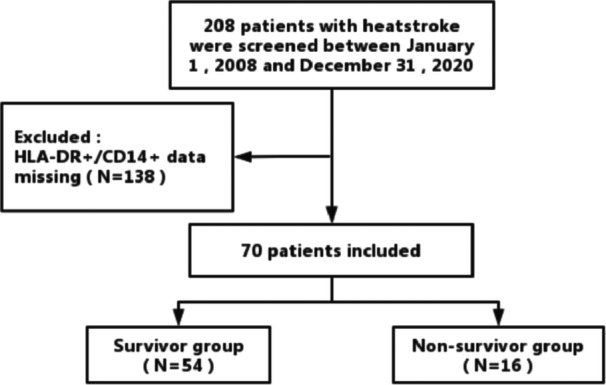
Flow diagram of inclusions and exclusions in exertional heatstroke patients. HLA‐DR, human leukocyte‐DR antigen.

The inclusion criterion was that patients aged ≥18 years old and met the diagnostic criteria for EHS. Patients were excluded as followed: (1) age < 18 years old; (2) patients not admitted within 48 h of EHS onset; (3) patients died within 24 h of admission; (4) pre‐existing tumors, severe organ dysfunction, or hematological system disorders; (5) pregnancy or breastfeeding; and (6) data missing, especially without mHLA‐DR measure in the first 24 h after admission. After gathering the clinical data of 281 patients and eliminating the missing data, 70 patients comprised of 54 survivors and 16 nonsurvivors were ultimately included (Figure 1).

### 2.3 Clinical data collection

Patients' information was collected from the digital medical record system. The month of onset, comorbidities, and general demographic data including age, gender, admission temperature (T), heart rate (HR), and so on. Clinical parameters, within 24 h of admission including blood routine, biochemistry, mHLA‐DR, and so on, and rhabdomyolysis (RM) were recorded. Disease severity scores including Glasgow coma scale (GCS) scores were recorded within 1 h at first medical service (at the site, on the ambulance, or at admission) before intubation and sedation. ICU and hospital stays' durations were recorded. The rapid and persistent cooling solutions were applied within 1 h at the first medical service (at the site, on the ambulance, or at admission). Patients were hospitalized within 48 h of onset, and blood analysis was performed within 1 h after admission.

### 2.4 Peripheral blood mHLA‐DR measure

Peripheral blood mHLA‐DR values were directly acquired from the database of the General Hospital of Southern Theatre Command of PLA in China. No images and figures could have been provided to show examples of representative cases. The percentage of HLA‐DR positive monocytes (%), not the mean fluorescence intensity (MFI), fluorescence unit relative to the monocyte population (RFU), and antibodies per cell (AB/c), was examined as the index of measured HLA‐DR via flow cytometer. According to operational guidelines for the clinical laboratory, standardly heparinized venous blood (2 mL) was incubated with fluorescein isothiocyanate (FITC) labeled anti‐HLA‐DR and phycoerythrin (PE) labeled anti‐CD14 monoclonal antibodies for 20 min under darkness and washed with phosphate buffer solution (PBS) three times. FITC and PE anti‐IgG1 monoclonal antibodies were explored to be the controls. The expression of CD14 served as a basis for categorization of monocytes. Results were presented as percentages of HLA‐DR‐positive monocytes relative to the overall population of monocytes. Each sample was examined for a minimum of 1500 monocytes. The peripheral blood mHLA‐DR level was tested on fixed days (Monday, Wednesday, or Friday) in our hospital, so individuals passed away within 24 h of admission and not be measured mHLA‐DR timely were excluded.

### 2.5 Statistical analysis

The Student's *t* tests or Mann–Whitney *U* tests were used appropriately to compare continuous variables (reported as means ± standard deviations or the medians and interquartile ranges) between groups. The Fisher's exact test or the *χ* ^2^ test was employed suitably to compare categorical data (summarized as counts and percentages). Logistic regression models were created to assess the correlation between potentially prognostic markers and in‐hospital death. The prediction model was represented as a nomogram, which was evaluated using calibration curves and a bootstrap of 1000 samples. Receiver operating characteristic (ROC) curves were created to assess the nomogram's effectiveness and independent risk factors, and their area under curves (AUCs) were acquired to assess the discriminative power. The group standards of systemic inflammatory response syndrome (SIRS), Acute Physiology and Chronic Health Evaluation (APACHE) II, and Sequential Organ Failure Assessment (SOFA) were divided according to the cutoff value of the highest Youden index. Net reclassification improvement (NRI) and integrated discrimination improvement (IDI) were counted to calculate the changes in assessment effectiveness of the prediction model and the comprehensive scores such as SIRS, SOFA, and APACHE II scores. Finally, with the net benefit rate as the vertical coordinate and the high‐risk threshold as the horizontal coordinate, the decision curve analysis (DCA) curve was constructed. Statistical analyses were performed using SPSS Windows version 27.0 (SPSS) and nomogram was developed using R software version 4.2.0. A two‐tailed *p* < 0.05 was considered statistically significant.

## 3 RESULTS

### 3.1 Baseline characteristics of EHS patients

**Figure 2:**
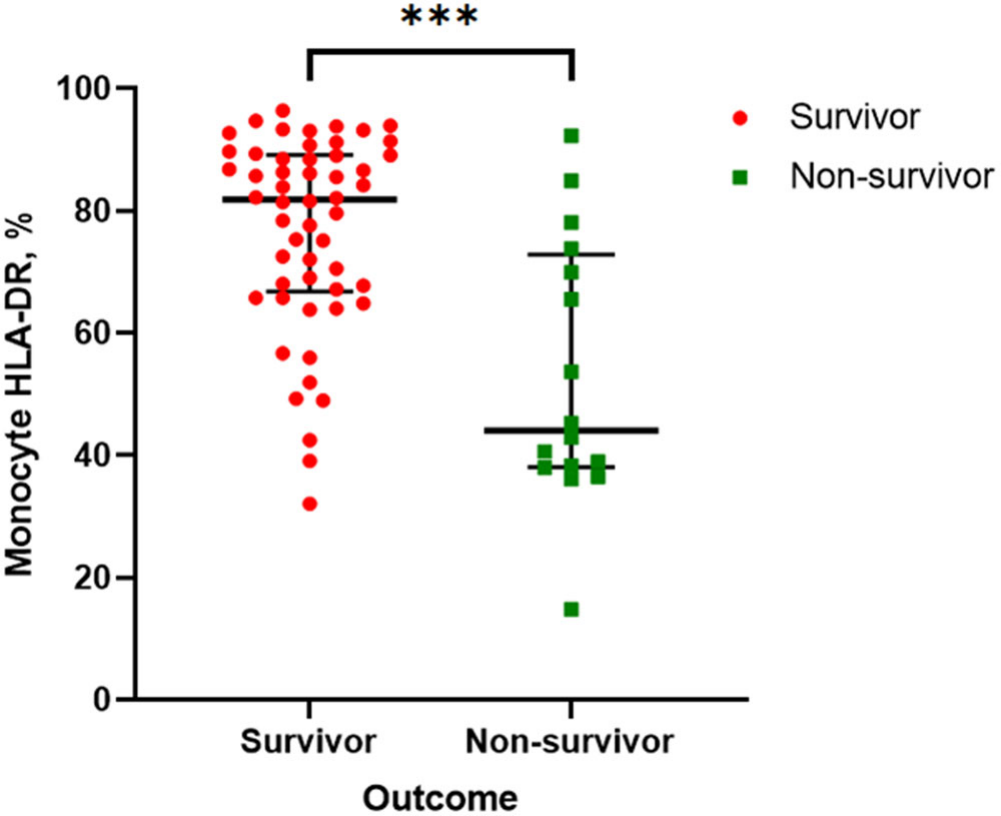
mHLA‐DR levels of EHS survivors versus nonsurvivors. EHS, exertional heatstroke; mHLA‐DR, CD14+ monocyte human leukocyte DR antigen level.

Seventy male patients comprised of 54 survivors and 16 nonsurvivors were ultimately analyzed. The patients' baseline demographic and clinical characteristics are shown in Table 1. The in‐hospital mortality rate was 22.86% (16/70). The levels of mHLA‐DR in the nonsurvivors (41.8% [38.1–68.1]%) were significantly lower than in the survivors (83.1% [67.6–89.4]%, *p* < 0.001) (Figure 2). APACHE II and SOFA scores in the nonsurvivors were significantly higher than those in the survivors (*p* < 0.001), reflecting that the disease severity of nonsurvivors is more serious. However, GCS scores were reversed between them (*p* < 0.001). Nonsurvivors (6 [37.5%]) require more vasoactive drugs than survivors (2 [3.7%], [*p* = 0.001]). The admission temperature, HR, aspartate aminotransferase, d‐dimer, prolonged prothrombin time, and international normalized ratio (INR) of nonsurvivors were higher than those of survivors (*p* < 0.05), which indicated worse underlying coagulation disorder and hepatic dysfunction in nonsurvivors. Besides, platelet (PLT), hemoglobin (Hb), and hematocrit (HCT) levels were lower in nonsurvivors (*p* < 0.05), indicating possible massive destruction of red blood cells in nonsurvivors. Furthermore, the length of ICU stays and hospital stays in those nonsurvivors (16 [9–20] days and 18 [9–46] days) were both longer than those in survivors (5 [3–8] days, *p* < 0.001 and 11 [4–22] days, *p* = 0.045). Mean arterial pressure, white blood cell, neutrophil count and percentage, prolonged activated partial thromboplastin time, fibrinogen, serum creatinine, creatine kinase, lymphocyte count and percentage, SIRS and International Society of Thrombosis and Hemostasis score, underlying disease, monocyte count and percentage, and RM between survivors and nonsurvivors were not significantly different.

**Table 1:** Baseline characteristics of EHS survivors versus nonsurvivors.

	Total (*N* = 70)	Survivor (*N* = 54)	Nonsurvivor (*N* = 16)	*p* Value
Age, years	23 (19–29)	22 (19–28)	27 (20–42)	0.192
Month				0.914
June, *N* (%)	22 (31.4%)	18 (33.3%)	4 (25.0%)	
July, *N* (%)	14 (20.0%)	10 (18.5%)	4 (25.0%)	
August, *N* (%)	10 (14.3%)	8 (14.8%)	2 (12.5%)	
Other, *N* (%)	24 (34.3%)	18 (33.3%)	6 (37.5%)	
Admission temperature, °C	37.2 (36.7–38.0)	37.0 (36.7–37.8)	37.9 (37.0–38.3)	0.010
HR, bpm	94 ± 24	87 ± 21	112 ± 26	<0.001
MAP, mmHg	87 ± 14	86 ± 12	90 ± 20	0.458
WBC, ×10^9^/L	11.45 ± 4.58	11.65 ± 4.61	10.78 ± 4.56	0.507
Neutrophil, ×10^9^/L	9.70 ± 4.26	9.82 ± 4.32	9.29 ± 4.16	0.666
Neu%, %	85.4 (80.3–90.1)	85.1 (80.8 −89.1)	88.3 (80.2–91.4)	0.211
Monocytes, ×10^9^/L	0.49 (0.28–0.86)	0.48 (0.30–0.86)	0.63 (0.18–0.79)	0.634
Mono%, %	4.8 (3.01–7.2)	4.6 (3.1–7.3)	5.4 (3.6–6.3)	0.939
Lymphocyte, ×10^9^/L	0.87 (0.40–1.53)	1.00 (0.56–1.56)	0.43 (0.30–1.17)	0.108
Lym%, %	8.2 (4.8–12.6)	8.7 (5.0–12.3)	7.1 (2.3–12.0)	0.213
Hb, g/L	130 ± 29	136 ± 22	110 ± 41	0.027
HCT, %	38.5 ± 8.2	40.1 ± 6.1	33.1 ± 11.9	0.036
PLT, ×10^9^/L	111 (45–179)	133 (56–182)	56 (23–112)	0.010
APTT, s	39.3 (33.6–45.3)	39.1 (33.7–41.9)	55.1 (31.1–104.2)	0.147
PT, s	17.7 (15.2–26.2)	16.1 (15.0–22.5)	26.2 (18.0–35.6)	0.002
INR	1.43 (1.20–2.39)	1.30 (1.17–1.88)	2.42 (1.46–3.68)	0.001
d‐dimer, μg/mL	3.39 (0.65–11.81)	1.32 (0.50–6.49)	11.52 (5.77–20.00)	<0.001
Fib, g/L	2.3 (1.8–2.7)	2.3 (1.9–2.7)	2.1 (1.2–2.7)	0.397
Scr, μmol/L	129 (91–179)	108 (87–138)	202 (160–317)	0.179
AST, U/L	126 (48–763)	81 (41–285)	244 (163–1810)	0.010
CK, U/L	1162 (520–4158)	1077 (495–3148)	1852 (1098–5474)	0.179
DIC, *N* (%)	27 (38.6%)	15 (27.8%)	12 (75.0%)	<0.001
Rhabdomyolysis, *N* (%)	40 (57.1%)	28 (51.8%)	12 (75.0%)	0.100
Vasoactive drug, *N* (%)	8 (11.4%)	2 (3.7%)	6 (37.5%)	0.001
Underlying disease, *N* (%)	8 (11.4%)	6 (11.1%)	2 (12.5%)	1.000
GCS score	14 (6–15)	15 (11–15)	4 (3–7)	<0.001
SIRS score	1 (1–2)	3 (2–5)	9 (7–11)	0.061
APACHE II score	10 ± 8	8 ± 6	20 ± 6	<0.001
SOFA score	4 (2–7)	3 (2–5)	9 (7–11)	<0.001
ISTH score	3 (2–6)	3 (2–4)	6 (4–7)	0.001
Length of ICU, days	7 (4–12)	5 (3–8)	16 (9–20)	<0.001
Length of Hospital, days	11 (5–27)	11 (4–22)	18 (9–46)	0.045
mHLA‐DR, %	76.5 (56.5–88.4)	83.1 (67.6–89.4)	41.8 (38.1–68.1)	<0.001

*Note*: All the above indicators were first measured within 24 h of onset.

Abbreviations: APACHE, Acute Physiology and Chronic Health Evaluation; APTT, activated partial thromboplastin time; AST, aspartate aminotransferase; CK, creatine kinase; DIC, disseminated intravascular coagulation; EHS, exertional heatstroke; Fib, fibrinogen; GCS, Glasgow Coma Scale; Hb, hemoglobin; HCT, hematocrit; HR, heart rate; INR, international normalized ratio; ISTH, International Society of Thrombosis and Hemostasis; Lym%, lymphocyte percent; MAP, mean arterial pressure; mHLA‐DR, CD14+ monocyte human leukocyte DR antigen level; Neu%, neutrophil percent; PLT, platelet; PT, prothrombin time; Scr, serum creatinine; SIRS, Systemic Inflammatory Response Syndrome; SOFA, Sequential Organ Failure Assessment; WBC, white blood cell.

### 3.2 mHLA‐DR expression independently predicting in‐hospital mortality

The prognostic‐relevant parameters identified by univariate logistic regression model are shown in Table 2. Poor outcomes of EHS patients were substantially correlated with admission temperature, HR, Hb, HCT, PLT, INR, GCS scores, vasoactive drug, mHLA‐DR, length of hospital stays and the presence of DIC (all *p* < .05). It is noteworthy that mHLA‐DR level was passively correlated with in‐hospital mortality rate (odds ratio [OR] = 0.917, 95% confidence interval [CI], 0.880–0.956, *p* < 0.001). Multivariate logistic regression analysis showed that mHLA‐DR (OR = 0.939; 95% CI: 0.892–0.988; *p* = 0.016) and GCS scores (OR = 0.726; 95% CI: 0.591–0.892; *p* = 0.002) were independent risk factors of poor prognosis (Table 3).

**Table 2:** Univariate logistic analysis of risk factors of in‐hospital mortality rate in EHS patients.

Variable	OR	95% CI	*p* Value
Admission temperature, °C	2.651	1.287–5.459	0.008
HR, bpm	1.049	1.018–1.080	0.002
Hb, g/L	0.970	0.950–0.991	0.005
HCT, %	0.903	0.840–0.971	0.006
PLT, ×10^9^/L	0.990	0.982–0.999	0.028
PT, s	1.033	0.995–1.071	0.087
INR	2.408	1.321–4.389	0.004
d‐dimer, μg/mL	1.029	0.996–1.063	0.083
AST, U/L	1.000	1.000–1.000	0.195
DIC, *N* (%)			
No			
Yes	7.800	2.172–28.017	0.002
Vasoactive drug, *N* (%)			
No			
Yes	15.600	2.745–88.659	0.002
GCS score	0.653	0.534–0.799	<0.001
Length of ICU, days	1.210	1.078–1.358	0.001
Length of hospital, days	1.005	0.988–1.022	0.571
mHLA‐DR, %	0.917	0.880–0.956	<0.001

Abbreviations: AST, aspartate aminotransferase; CI, confidence interval; DIC, disseminated intravascular coagulation; EHS, exertional heatstroke; GCS, Glasgow Coma Scale; Hb, hemoglobin; HCT, hematocrit; HR, heart rate; ICU, intensive care unit; INR, international normalized ratio; mHLA‐DR, CD14+ monocyte human leukocyte DR antigen level; OR, odds ratio; PLT, platelet; PT, prothrombin time.

**Table 3:** Multivariate logistic analysis of risk factors of in‐hospital mortality rate in EHS patients.

Variable	*β*	OR	95% CI	*p* Value
mHLA‐DR	−0.063	0.939	0.892–0.988	0.016
GCS score	−0.320	0.726	0.591–0.892	0.002
Constant	5.645	282.816		0.001

*Note*: *β* is the regression coefficient, and *p* value < 0.05 means significant.

Abbreviations: CI, confidence interval; EHS, exertional heatstroke; GCS, Glasgow Coma Scale; mHLA‐DR, CD14+ monocyte human leukocyte DR antigen level; OR, odds ratio.

### 3.3 Construction of the nomogram based on mHLA‐DR in EHS patients

**Figure 3:**
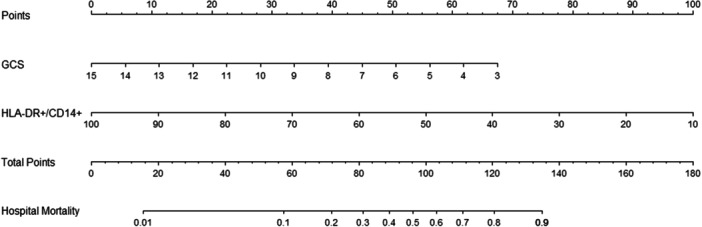
The nomogram of the prediction model. GCS, Glasgow Coma Scale; mHLA‐DR, CD14+ monocyte human leukocyte DR antigen level.

**Figure 4:**
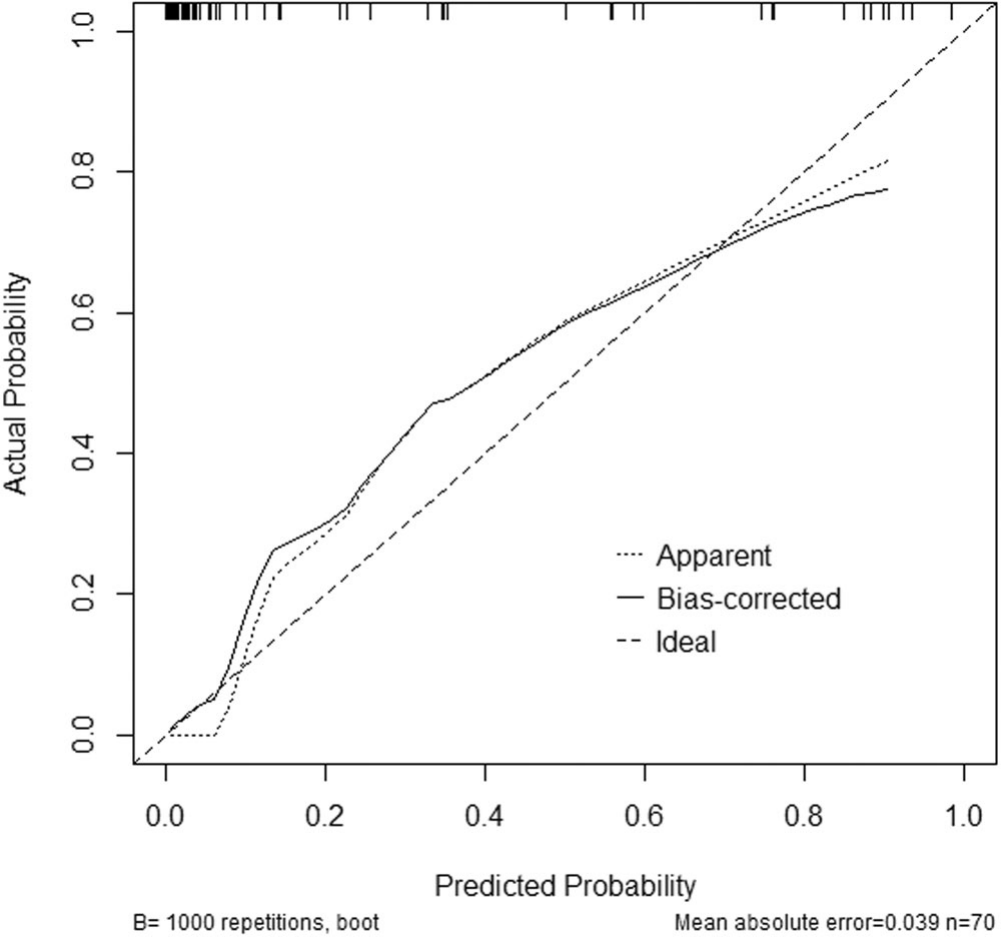
The calibration curves of the nomogram.

A nomogram was created to predict the mortality rate for EHS patients based on mHLA‐DR and GCS score (Figure 3). The final scores were calculated by adding mHLA‐DR to the GCS score, which was utilized for predicting the inpatients' mortality. The calibration curve indicated that the nomogram performed well in predicting hospital mortality, which was consistent with actual probabilities (*p* = 0.483 > 0.05) (Figure 4).

### 3.4 Prediction model based on mHLA‐DR and GCS score superior to scoring systems for evaluating prognosis in EHS patients

We further integrated mHLA‐DR and GCS score into the prediction model of in‐hospital mortality rate as follows:

*Z* = Logit (ODDs) = 5.645 – 0.063 × (mHLA‐DR) − 0.320 × GCS score (Model 1).

*Note*: ODDs = ln (*P*/1 − *P*), *P* = e^*Z*/(1 + e^*Z*).

**Figure 5:**
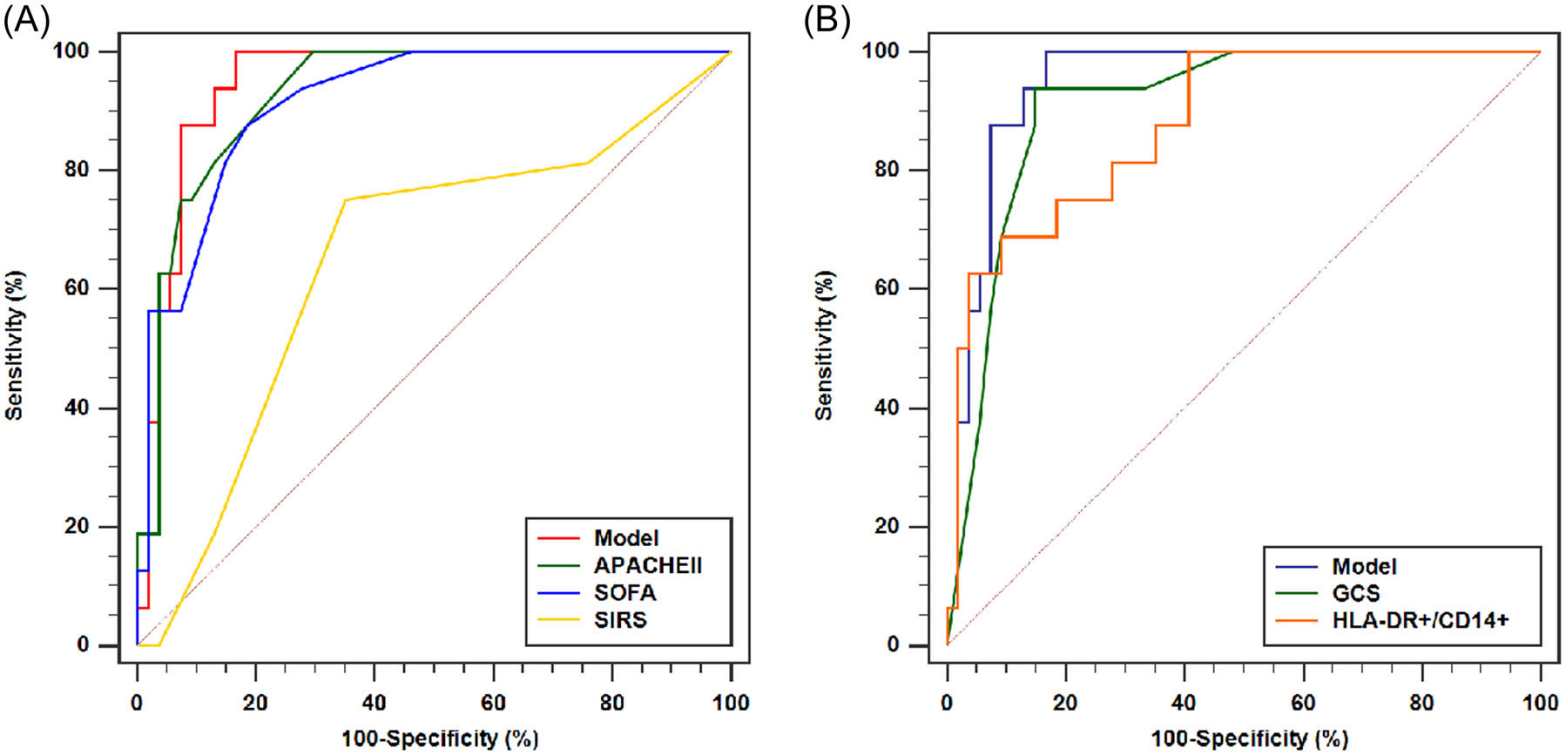
Comparison of ROC curves between independent prognostic factors and models. (A) Comparison of ROC curves between model, GCS, and mHLA‐DR. (B) Comparison of ROC curves between model, SOFA, APACHE II, and SIRS. APACHE II, acute physiology and chronic health valuation II; GCS, Glasgow Coma Scale; mHLA‐DR, CD14+ monocyte human leukocyte DR antigen level; ROC, receiver operating characteristic; SIRS, systemic inflammatory response syndrome; SOFA, sequential organ failure assessment.

The outcomes of the Hosmer–Lemeshow test showed that the prediction model successfully suited the data (*χ* ^2^ = 7.512, *df* = 8, *p* = 0.483). As shown in Figure 5A and Table 4, the AUC value for the prediction model was 0.947, with a 95% confidence interval of 0.865–0.986 with a sensitivity of 100.00% and a specificity of 83.33%. Interestingly, this value did not differ significantly from the AUC value of the two independent prognostic factors, mHLA‐DR (AUC = 0.880, 95% CI: 0.780–0.945, *p* = 0.0714) and GCS (AUC = 0.910, 95% CI: 0.817–0.965, *p* = 0.0939).

**Table 4:** Comparison of ROCs between independent prognostic factors and models.

	AUC	95% CI	*p* Value	Cutoff	SEN (%)	SPE (%)	YI
Model	0.947	0.865–0.986	<0.0001	0.1253	100.00	83.33	0.8333
GCS score	0.910	0.817–0.965	<0.0001	8	93.75	85.19	0.7894
mHLA‐DR	0.880	0.780–0.945	<0.0001	53.6	68.75	90.74	0.5949
SOFA	0.916	0.825–0.969	<0.0001	5	87.50	81.48	0.6898
APACHE II	0.929	0.841–0.977	<0.0001	10	100.00	70.37	0.7037
SIRS	0.649	0.526–0.760	0.0625	1	75.00	64.81	0.3981

Abbreviations: APACHE II, acute physiology and chronic health valuation II; AUC, area under the curve; CI, confidence interval; GCS, Glasgow Coma Scale; mHLA‐DR, CD14+ monocyte human leukocyte DR antigen level; ROCs, receiver operating characteristics; SEN, sensitivity; SIRS, systemic inflammatory response syndrome; SOFA, sequential organ failure assessment; SPE, specificity; YI, Youden Index.

As shown in Figure 5B and Table 4, the prediction model's AUC was not significantly different from APACHE II (0.929, *p* = 0.4904) and SOFA scores (0.916, *p* = 0.3701), it was considerably higher than the SIRS score's AUC (0.649, *p* = 0.0003) (Table 4). The prediction model was shown with a superior specificity (83.33%), in contrast to SOFA (81.48%), APACHE II (70.37%), and SIRS (64.81%) scores. The NRI values were 1.5278 (95% CI: 1.1629–1.8927, *p* < 0.0001), 0.2963 (95% CI: −0.2560 to 0.8486) and 0.7685 (95% CI: 0.2421–1.2949) when the prediction model was compared to the SIRS score, APACHE II and SOFA score, and the corresponding IDI values were 0.5148 (95% CI: 0.3671–0.6642, *p* < 0.0001), 0.0659 (95% CI: −0.1148 to 0.2465), and 0.0892 (95% CI: −0.0411 to 0.2195), indicating the better prognosis efficiency of the prediction model (Supporting Information S1: Table S1).

### 3.5 Prediction model superior to scoring systems for clinical decision‐making in EHS patients

**Figure 6:**
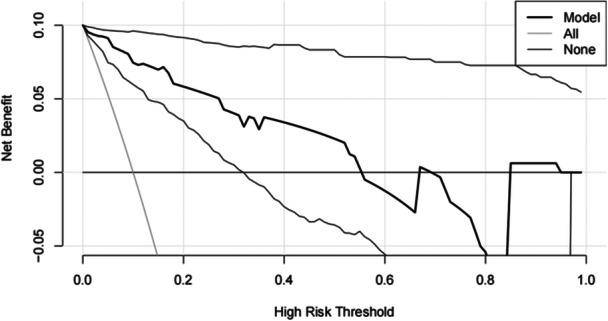
Decision curve analysis and confidence interval curve of model.

A DCA curve was created to demonstrate the prediction model's clinical applicability and compared with traditional scoring systems. The prediction model with the DCA curve being on the top when the high‐risk threshold was approximately 0–0.9 is superior to the comprehensive scoring system in clinical decision‐making (Supporting Information S1: Figure S1). Clinical interventions led by the prediction model showed a larger net benefit and a higher application value than the scoring systems. For paired model comparisons at each of these thresholds, two curves were drawn to indicate the 95% CI for the difference in net benefit, presenting confidence intervals for a decision curve analysis (Figure 6).

## 4 DISCUSSION

This study investigated the relationship between mHLA‐DR and the prognosis in EHS patients and the key findings were as follows: (1) mHLA‐DR levels within 24 h after admission were significantly lower in the nonsurvivors. (2) mHLA‐DR and GCS score were independent risk factors for in‐hospital mortality. (3) The nomogram integrated mHLA‐DR and GCS score, with better predictive performance and higher net benefits than traditional scoring systems, was established to predict mortality rate in EHS patients.

One of the areas of current research of EHS focuses mainly on exploring novel biomarkers that can more accurately prognosing short‐ and long‐term outcomes.^6^ By far, no study has been carried out to explore whether mHLA‐DR is associated with the prognosis in EHS patients. This study revealed that mHLA‐DR was an independent risk factor (OR = 0.939; 95% CI: 0.892–0.988; *p* = 0.016) for poor outcomes in EHS patients. We found that mHLA‐DR levels had a modest prognostic effect for in‐hospital mortality rate (AUC = 0.880, 95% CI: 0.780–0.945, cut‐off value = 53.6%, *p* < 0.0001) in EHS, which was consistent with the previous studies illustrating that decreased mHLA‐DR expression predicted poor outcomes in patients with severe burns,^20^ and sepsis.^15^ Berres et al. have described that individuals with decompensated liver cirrhosis had greater ICU mortality rate when their mHLA‐DR levels were decreased.^10^ Monneret et al. demonstrated that lower mHLA‐DR levels had worse survival rates in sepsis patients.^21^

The clinical characteristics of EHS are comparable to sepsis,^22^ indicating that immune disorder takes key roles in EHS possibly. The immune disturbances mainly manifest as the suppression of immune cells and their functions.^23^ In vitro and in vivo studies to date have shown that prolonged exposure to a febrile range may reduce monocyte bactericidal capacity and cytokine production.^24^ Downregulation of mHLA‐DR, as one of the diagnostic indices for immune paralysis, is a crucial component of monocyte deactivation in critically ill individuals,^25^ which is correlated with the disease severity at admission.^26^

Decreased expression of HLA‐DR makes monocytes unable to present the antigen information to the immune effector cells to trigger an efficient immune response and clear the pathogen effectively. Excessive amounts of inflammatory mediators and cytokines are released from types of activated cells, resulting in inflammatory cascade effect and abnormal monocyte function in EHS patients, including interleukin‐6 (IL‐6), tumor necrosis factor‐α (TNF‐α) and platelet‐activating factor (PAF).^27^ One of these manifestations is the decreased expression of mHLA‐DR, which can possibly cause immune suppression and accelerate the development of sepsis.^28^ Elevated levels of glucocorticoids^29^ and interleukin‐10 (IL‐10),^30^ which have been reported in EHS patients,^1^, ^31^ may be a potential mediator to down‐regulate mHLA‐DR level.

As the most commonly susceptible to heat stress, CNS injury is one of the most serious complications of EHS.^32^ Some clinical studies have shown that neurological disorders are negatively correlated to the advanced mortality rate of ICU individuals with heat‐related diseases.^33^ GCS score is a scoring system for specific organs or diseases used to assess the severity of CNS damage.^34^ The current study demonstrated GCS scores (OR = 0.726; 95% CI: 0.591–0.892; *p* = 0.002) was independent risk factors related with in‐hospital mortality rate in EHS. HS can induce not only direct cytotoxicity to CNS and cerebral ischemia hypoxia caused by decreased cardiac output, but also secondary inflammatory injury to the CNS.^35^ A significant therapeutic purpose might be to increase the GCS score through protecting and cooling the brain.^36^ Early aggressive treatment of these potential risk factors may help restore CNS function in patients with EHS.

It has been shown that CNS injury can directly induce immunodepression.^37^, ^38^, ^39^, ^40^ Circulating monocytes from patients with acute brain injuries have decreased expression of major histocompatibility complex (MHC) class II,^38^, ^40^ and impaired monocyte functions resulting in insufficient antigen‐presentation and decreased expression of secreted or membrane‐bound costimulatory molecules.^39^ Given CNS injury is an early event, it is hypothesized that CNS injury directly induced monocyte dysfunction. Therefore, it is reasonable to employ this model based on mHLA‐DR and GCS score as a prognosis candidate in EHS. The prediction model constructed on mHLA‐DR and GCS score showed a good predictive performance for in‐hospital mortality rate (AUC = 0.947, 95% CI: 0.865–0.986, *p* < 0.0001). The SOFA and APACHE II scores, not specifically for EHS, are frequently employed in the severity assessment of critical illness.

The prediction model of this study included mHLA‐DR and GCS score, which reflected the important pathophysiological changes in the immune and CNS of patients with EHS, had a higher specificity and better predictive performance (SEN = 100.00%, SPE = 83.33%, NRI > 0, IDI > 0). Compared with other prognostic scoring systems, the prediction model contains fewer variables and is easy to obtain clinically. Additionally, the prediction model with the DCA curve being on the top when the high‐risk threshold was approximately 0–0.9 is superior to the comprehensive scoring system in clinical decision‐making. Summarily, the prediction model was possibly more suitable and practical for grading diagnosis, guiding treatment, and judging prognosis in patients with EHS. However, it is certainly necessary to further evaluate the predictive efficiency of the model by rigorous internal and external validation before clinical application.

## 5 LIMITATIONS

Firstly, the sample size for this single‐center retrospective study was small, which could possibly result in a statistical bias. Secondly, the patients were mostly young males, with fewer females, so caution should be exercised when extending the results to the entire public. Thirdly, although the method to measure mHLA‐DR in our lab had been reported in many studies,^41^, ^42^, ^43^ it was possibly not as same standardized as value measured as “anti HLA‐DR antibody molecules that were bound per monocyte.”^44^, ^45^ Finally, this study analyzed the difference in mHLA‐DR within 24 h after EHS onset comparing between the survivors and nonsurvivors, and no dynamic changes of mHLA‐DR were analyzed, which poorly pictured the kinetic of mHLA‐DR poorly.

## 6 CONCLUSION

mHLA‐DR within 24 h after admission and GCS score were the independent risk factors for in‐hospital mortality rate in EHS patients, indicating immune paralysis and CNS damage were the key intervention targets clinically. The nomogram based on mHLA‐DR and GCS score, with better predictive performance and higher net benefits than traditional scoring systems, was possibly more suitable and practical for grading diagnosis, guiding treatment, and judging prognosis in EHS patients. More research was required to verify whether mHLA‐DR could be an ideal biomarker for forecasting short‐ and long‐term outcomes and guiding treatment in EHS patients.

## AUTHOR CONTRIBUTIONS

**Fanfan Wang**: Data curation; manuscript preparation; manuscript review; manuscript editing. **Fanghe Gong**: Conceptualization. **Xuezhi Shi**: Data curation; formal analysis. **Jiale Yang**: Formal analysis; methodology. **Jing Qian**: Investigation. **Lulu Wan**: Visualization. **Huasheng Tong**: Funding acquisition; supervision; manuscript review; manuscript editing.

## CONFLICT OF INTEREST STATEMENT

The authors declare no conflict of interest.

## ETHICS STATEMENT

The study protocol was confirmed by the Medical Ethics Review Committee of the General Hospital of Southern Theater of PLA of China (No. NZLLKZ2022047), and the demand for informed authorization was eliminated.

## Supporting information

Supporting information.

## Data Availability

The data that support the findings of this study are available from the corresponding author, (Huasheng Tong), upon reasonable request.
