# Supplementary material for: Monocyte HLA‐DR level on admission predicting in‐hospital mortality rate in exertional heatstroke: A 12‐year retrospective study
Source: Immun Inflamm Dis. 2024 Apr 17;12(4):e1240. doi: 10.1002/iid3.1240 (PMC11022625; doi:10.1002/iid3.1240)
Supplement: Supplementary file 1 — Supporting information. [file IID3-12-e1240-s001.docx]

**Supplementary Material**

**Title manuscript:** Monocyte HLA-DR level on admission predicting in-hospital mortality in exertional heatstroke: a 12-year retrospective study

**Authors**

Fanfan Wang^1,3^, Fanghe Gong^2^, Xuezhi Shi^3^, Jiale Yang^3^, Jing Qian^3^, Lulu Wan^3^, Huasheng Tong^1,3*^

**Affiliations**

^1^ The First School of Clinical Medicine, Southern Medical University, Guangzhou 510000, China

^2^ Department of Neurosurgery, General Hospital of Southern Theatre Command of PLA, Guangzhou 510010, China

^3^ Department of Intensive Care Unit, General Hospital of Southern Theatre Command of PLA, Guangzhou 510010, China

* Corresponding author

Huasheng Tong, Department of Intensive Care Unit, General Hospital of Southern Theatre Command of PLA, Guangzhou, 510000, China. Email: fimmuths@163.com; Tel: (86) 13710960796.

**Content of Supplementary Material**

**Supplementary Tables**

- Table S1 Page 3

**Supplementary Figures**

- Figure S1 Page 4

**Supplementary Tables**

Table S1 Comparison of NRI and IDI between Model, SOFA, APACHE Ⅱ and SIRS

| Old model |  | | 95%CI | P-value |
| --- | --- | --- | --- | --- |
| SIRS | NRI | 1.5278 | 1.1629-1.8927 | <0.0001 |
|  | IDI | 0.5148 | 0.3671-0.6624 | <0.0001 |
| APACHE II | NRI | 0.2963 | -0.2560-0.8486 | 0.2930 |
|  | IDI | 0.0659 | -0.1148-0.2465 | 0.4747 |
| SOFA | NRI | 0.7685 | 0.2421-1.2949 | 0.0042 |
|  | IDI | 0.0892 | -0.0411-0.2195 | 0.1798 |

NRI, net reclassification improvement; IDI, integrated discrimination improvement; SOFA, sequential organ failure assessment; APACHE Ⅱ, acute physiology and chronic health valuation Ⅱ; SIRS, systemic inflammatory response syndrome; CI, confidence interval; p-value <0.05 means significant.

**Supplementary Figures**


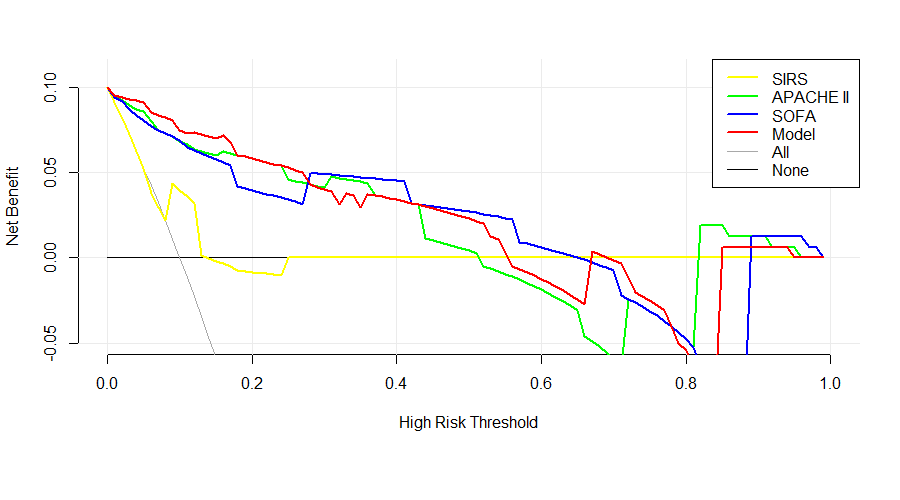


Figure S1 DCA of Model, SOFA, APACHE Ⅱ and SIRS. SOFA, sequential organ failure assessment; APACHE Ⅱ, acute physiology and chronic health valuation Ⅱ; SIRS, systemic inflammatory response syndrome ; horizontal line (no treatment intervention for all patients); diagonal line (treatment intervention for all patients).
